# Ivermectin has New Application in Inhibiting Colorectal Cancer Cell Growth

**DOI:** 10.3389/fphar.2021.717529

**Published:** 2021-08-13

**Authors:** Shican Zhou, Hang Wu, Wenjuan Ning, Xiao Wu, Xiaoxiao Xu, Yuanqiao Ma, Xingwang Li, Junhong Hu, Chenyu Wang, Junpeng Wang

**Affiliations:** Infection and Immunity Institute and Translational Medical Center of Huaihe Hospital, Henan University, Kaifeng, China

**Keywords:** ivermectin, colorectal cancer, oxidative stress, apoptosis, cell cycle

## Abstract

Colorectal cancer (CRC) is the third most common cancer worldwide and still lacks effective therapy. Ivermectin, an antiparasitic drug, has been shown to possess anti-inflammation, anti-virus, and antitumor properties. However, whether ivermectin affects CRC is still unclear. The objective of this study was to evaluate the influence of ivermectin on CRC using CRC cell lines SW480 and SW1116. We used CCK-8 assay to determine the cell viability, used an optical microscope to measure cell morphology, used Annexin V-FITC/7-AAD kit to determine cell apoptosis, used Caspase 3/7 Activity Apoptosis Assay Kit to evaluate Caspase 3/7 activity, used Western blot to determine apoptosis-associated protein expression, and used flow cytometry and fluorescence microscope to determine the reactive oxygen species (ROS) levels and cell cycle. The results demonstrated that ivermectin dose-dependently inhibited colorectal cancer SW480 and SW1116 cell growth, followed by promoting cell apoptosis and increasing Caspase-3/7 activity. Besides, ivermectin upregulated the expression of proapoptotic proteins Bax and cleaved PARP and downregulated antiapoptotic protein Bcl-2. Mechanism analysis showed that ivermectin promoted both total and mitochondrial ROS production in a dose-dependent manner, which could be eliminated by administering N-acetyl-l-cysteine (NAC) in CRC cells. Following NAC treatment, the inhibition of cell growth induced by ivermectin was reversed. Finally, ivermectin at low doses (2.5 and 5 µM) induced CRC cell arrest. Overall, ivermectin suppressed cell proliferation by promoting ROS-mediated mitochondrial apoptosis pathway and inducing S phase arrest in CRC cells, suggesting that ivermectin might be a new potential anticancer drug therapy for human colorectal cancer and other cancers.

## Introduction

Colorectal cancer (CRC) refers to malignant tumors in the ascending colon, transverse colon, descending colon, sigmoid colon, and rectum and is one of the most common malignant tumors worldwide. Among all malignant tumors globally, CRC ranks third in incidence and second in mortality (Siegel et al., 2020). CRC has caused a heavy economic burden on the country and individuals (Maida et al., 2017). At present, the treatment of CRC mainly adopts a comprehensive treatment based on surgery, combined with radiotherapy, chemotherapy, targeted therapy, and other treatments (Modest et al., 2019). However, due to the complicated mechanism of the occurrence, development, and metastasis of CRC, there is still a lack of specific drugs for CRC treatment.

Ivermectin is a derivative of the 16-membered macrolide compound abamectin, which was first widely used in clinical practice as an antiparasitic drug (Laing et al., 2017). Ivermectin can increase the activity of γ-aminobutyric acid receptor or glutamate-chloride ion channel (Glu-Cl), increase the influx of chloride ions, and cause the cell membrane hyperpolarization, thereby blocking signal transmission between neurons and muscles (Martin et al., 2021), which exerts its antiparasitic effects. Ivermectin could be used, in addition to as an antiparasitic drug, as antiviral agents such as Flavivirus, HIV-1 virus, and SARS-CoV-2 virus (Mastrangelo et al., 2012; Wagstaff et al., 2012; Caly et al., 2020). Moreover, studies have shown that ivermectin has an inhibitory effect on various tumor cells and may be a potential broad-spectrum antitumor drug (Juarez et al., 2020). Juarez et al. (2020) have demonstrated that ivermectin is the most sensitive to breast cancer cells MDA-MB-231, MDA-MB-468, MCF-7, and ovarian SKOV-3; whereas ivermectin is the most nonsensitive to the prostate cancer cell line DU145. The induction of cell cycle arrest at G0/G1 mediates this effect of ivermectin on these sensitive cancer cells. Furthermore, ivermectin can inhibit the proliferation of cancer cells through p21-activated kinase 1 (PAK1)-induced autophagy, Caspase-dependent apoptosis, or immunogenic cell death regulate the signal pathways, including Hippo, Akt/mTOR, and WNT-TCF pathways to inhibit cancer cell proliferation (Liu et al., 2020). As known, ROS plays a vital role in the apoptosis caused by oxidative stress. ROS is a by-product of normal mitochondrial respiration. Stimuli such as infection, drought, cold, and ultraviolet light result in increased ROS in cells. Then, accumulative ROS could induce cells mitochondrial dysfunction and promote apoptosis in cells (Sinha et al., 2013). Evidence has shown that ivermectin-induced apoptosis is closely related to the production of ROS. Currently, there are few reports on the research of ivermectin in colorectal cancer.

Furthermore, new use of old drugs (that is, drug relocation) is a strategy for expanding old drugs and developing new uses, which has the advantages of low research and development cost and short development time (Pushpakom et al., 2019). Research on drug relocation of ivermectin is a shortcut to developing new antitumor drugs. Given this, we designed a study to explore the impact of ivermectin on the proliferation and apoptosis of CRC cells and the underlying mechanism.

## Materials and Methods

### Cell Culture

SW480 and SW1116 cells were acquired from ATCC and grown in DMEM medium (Biological Industries, Israel) supplemented with 10% FBS (Biological Industries, Israel), 1% penicillin/streptomycin (Coolaber, Beijing, China), and 2.5% HEPES buffer (Procell, Wuhan, China) in an incubator with a humidified air atmosphere of 5% CO_2_ at 37°C.

### Cell Viability Assay

**FIGURE 1:**
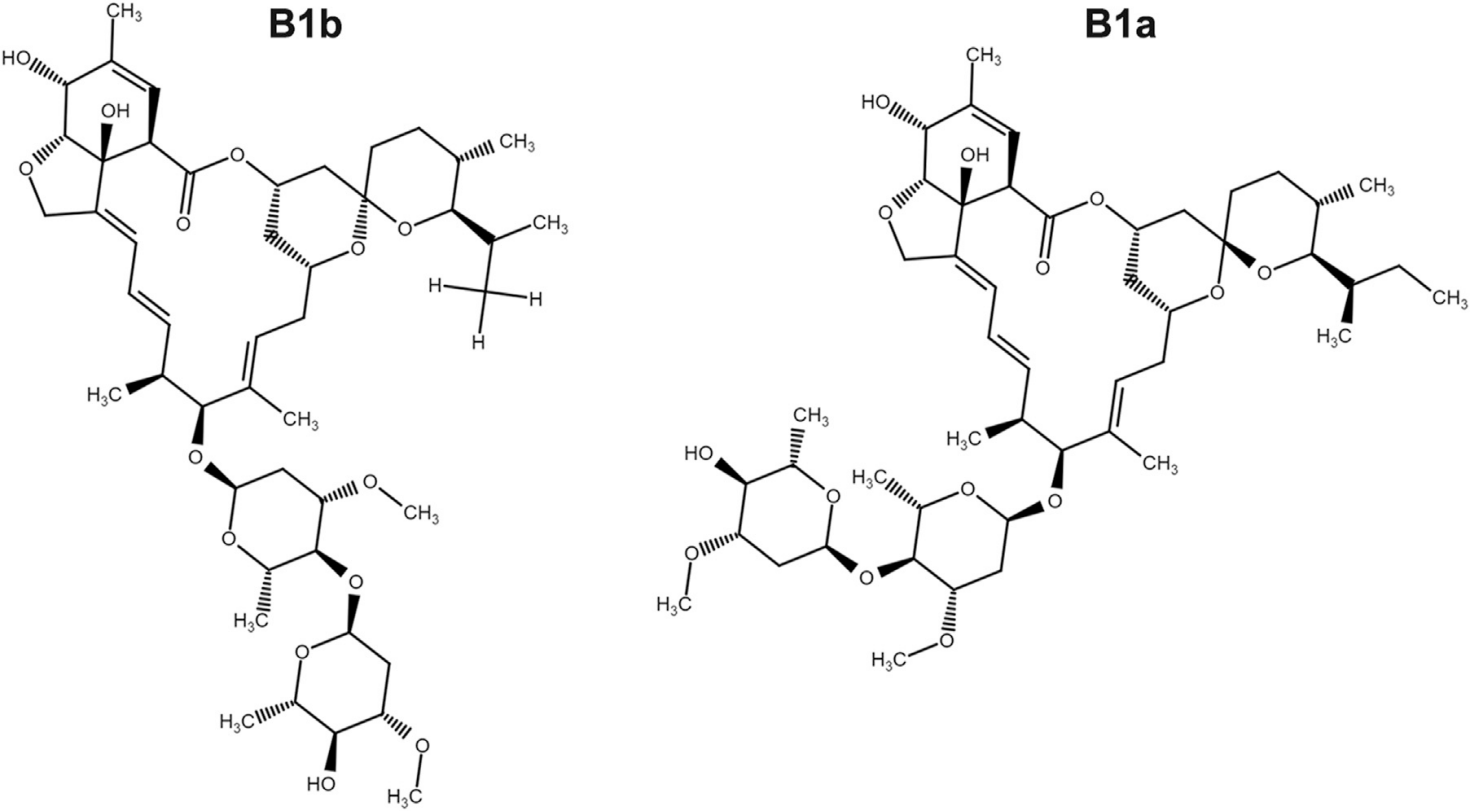
The chemical structure of ivermectin which is composed of ivermectin B1a (>90%) and ivermectin B1b.

Cells were seeded at a density of 1 × 10^4^ cells/well in a 96-well plate. After being cultured overnight, cells were treated with ivermectin (Figure 1) (MCE Chemicals, Shanghai, China) at the indicated concentrations for 12, 24, or 36 h or cells were pretreated with N-Acetyl-l-cysteine (NAC, 5 mM) (Aladdin, Shanghai, China) for 1 h and then were cultured in ivermectin (20 μM) for 6 h. Then, 10 μL of CCK-8 solution was added to each well and incubated at 37°C for 1 h. The absorbance was detected at 450 nm by a microplate reader (SpectraMax i3x, Molecular Devices, United States). The cell viability was calculated as follows: (absorbance of drug-treated sample/absorbance of control sample) × 100.

### Cell Morphology

Colorectal cancer cells were plated at 1 × 10^5^ cells/well in twelve-well plates. After being cultured overnight, the cells were treated with ivermectin at the indicated dose for 24 h. Cell morphology was evaluated using an optical microscope.

### Flow Cytometry

Apoptosis was determined using the Annexin V-FITC/7-AAD Apoptosis Kit. Briefly, after colorectal cancer cells were exposed to ivermectin (0, 5, 10, and 20 μM) for 6 h, cells were centrifuged at 1,500 rpm for 5 min, washed, and suspended in PBS. Then, cells were stained with Annexin V-FITC and 7-AAD for 15 min. In addition, cells (1 × 10^6^ cells/well) were cultured onto 6-well plates overnight and treated with indicated concentrations (0, 2.5, 5, and 10 μM) of ivermectin. Then, the cells were harvested and resuspended in PBS and fixed with 70% ethanol, and left at −20°C overnight. After 12 h of fixation, cells were centrifuged, washed, and resuspended in cold PBS. Then, the cells were added with 100 μL of RNase and incubated at 37°C for 30 min. The PI staining solution was then added and incubated at 4°C for 30 min. Cells were acquired by flow cytometry (FACSCanto Plus), and data were analyzed using Flowjo 10.0 software. The percentage of Q2 (early apoptosis, Annexin V^+^7-AAD^-^) plus Q3 (late apoptosis, Annexin V^+^7-AAD^+^) region was counted as the percentage of apoptosis cells.

### Caspase 3/7 Activity Assay

Caspase 3/7 assay was performed using the Caspase 3/7 Activity Apoptosis Assay Kit (Sangon Biotech, Shanghai, China). Briefly, after colorectal cancer cells (SW480 or SW1116) were treated with different concentrations of ivermectin (0, 5, 10, and 20 μM) for 6 h, we added 100 μL of Caspase 3/7 reagent into each well and mixed using a plate shaker. The Caspase 3/7 activity was then determined using a microplate reader (SpectraMax i3x). The Caspase 3/7 activity was expressed as a fold of the untreated control (Con) treatment.

### Western Blot Assay

After colorectal cancer cells (SW480 and SW1116) were treated with 0, 5, and 10 μM ivermectin for 6 h, they were collected, washed with PBS, and lysed with RIPA buffer. Protein quantification was determined using BCA Protein Assay Kit (EpiZyme Biotechnology, Shanghai, China). Equal amounts of protein were loaded onto an SDS-PAGE gel for electrophoresis and transferred to nitrocellulose. After blocking with 5% nonfat milk for 1 h, the membranes were incubated with the primary antibody [Bcl-2 (1:2000), Bax (1:2000), PARP (1:20,000) (all from Proteintech, Rosemont, IL, United States), and β-actin (1:5000) (Sigma-Aldrich)] on a 4°C shaker overnight. The membranes were then incubated with a secondary antibody for another 1 h at room temperature. A chemiluminescent gel imaging system detected the change in target protein expression (Universal Hood II, Bio-Rad, Hercules, CA, United States).

### ROS Measurement

For total ROS measurement, colorectal cancer cells (SW1116) were seeded (1 × 10^5^ cells/well) in a 12-well plate and incubated overnight. Cells were treated with different concentrations of ivermectin for another 6 h, and then they were co-cultured with DCFH-DA (Invitrogen, Carlsbad, CA, United States) and DAPI (Biolegend, San Diego, CA, United States) for 20 min at 37°C in the dark. The cell fluorescence was photographed by fluorescence microscopy (OLYMPUS, Tokyo, Japan).

For the mitochondrial ROS measurement, colorectal cancer cells (SW1116) were seeded in a 12-well plate and incubated overnight. After that, cells were treated with 0, 2.5, 5, 10, and 20 μM ivermectin for another 6 h, and then they were tinted with oxidation of MitoSOX Red (Invitrogen, Carlsbad, CA) and DAPI, which is oxidized by superoxide in the mitochondria, emitting red fluorescence. Cultures were incubated for 10 min at 37°C and washed twice with warm HBSS. Production of mitochondrial ROS was analyzed using MitoSOX Red. The cell fluorescence was photographed by fluorescence microscopy.

### Data Analysis

All experiments were repeated at least three times, and data were presented as mean ± S.E.M. The statistics were analyzed using a one-way or two-way ANOVA analysis (ANOVA) followed by Tukey’s test using Prism 9.0 software (Graphpad Software). *p* values are *, *p* < 0.05, ^#^, *p* < 0.05;

## Results

### Ivermectin Inhibits the Proliferation of Colorectal Cancer Cells

**FIGURE 2:**
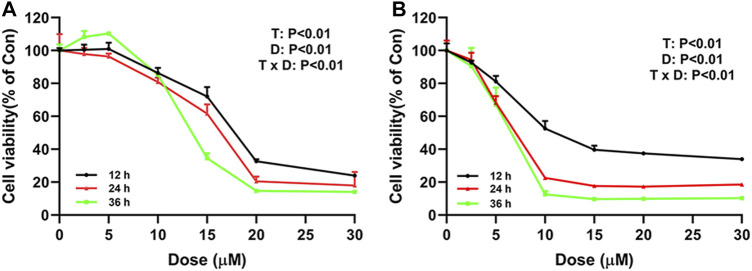
Ivermectin inhibits cell proliferation of SW480 and SW1116 cells. SW480 **(A)** and SW1116 cells **(B)** were cultured at different concentrations of ivermectin (0, 2.5, 5, 10, 15, 20, and 30 μM) for 12, 24, and 36 h, and then CCK-8 assay was performed to detect the cell proliferation. The experiment was repeated three times, and data were presented as mean ± S.E.M. The interaction between dose (D) and time (T) effect were analyzed using two-way ANOVA following Tukey’s *t*-test. D: dose effect; T: time effect: T × D: the interaction between time and dose effect.

To explore the effect of ivermectin on the proliferation of colorectal cancer cells, we used different concentrations of ivermectin (0, 2.5, 5, 10, 15, 20, and 30 μM) to culture colorectal cancer cells SW480 and SW1116. CCK-8 assay was performed to measure SW480 and SW1116 cancer cell proliferation after cells were incubated for 12, 24, and 36 h. As shown in Figure 2, the cell viability of SW480 and SW1116 cells decreased dose-dependently by ivermectin treatment (Dose, D: *p* < 0.01). Furthermore, ivermectin inhibited SW480 and SW1116 cell viability in a time-dependent manner (Time, T: *p* < 0.01). Finally, Table 1 showed that the IC_50_ of SW480 cells treated with ivermectin for 12, 24, and 36 h was 16.17 ± 0.76 μM, 15.34 ± 0.81 μM, and 12.11 ± 0.97 μM, respectively; and the IC_50_ of SW1116 cells treated with ivermectin for 12, 24, and 36 h were 7.60 ± 0.62 μM, 6.27 ± 0.70 μM and 5.76 ± 0.81 μM, respectively. The present data indicate that ivermectin might have more sensitive to SW1116 cells than that of SW480 cells.

**TABLE 1:** Inhibitory concentration (IC_50_) of IVM on the viability of colorectal cells.

Cell line	IC_50_(μM)		
	12 h	24 h	36 h
SW480	16.17 ± 0.76	15.34 ± 0.81	12.11 ± 0.97
SW1116	7.60 ± 0.62	6.27 ± 0.70	5.76 ± 0.81

Data are presented as the mean ± S.E.M.. IC_50_, concentration required for 50% inhibition.

### Ivermectin Changes the Morphology of Colorectal Cancer Cells

**FIGURE 3:**
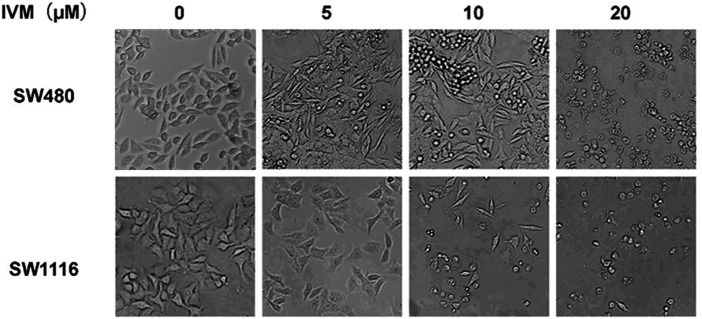
The effect of ivermectin on the morphology of colorectal cancer cells SW480 and SW1116 cells. Colorectal cancer SW480 **(A)** and SW1116 **(B)** cells were treated with different concentrations of ivermectin (0, 5, 10, and 20 μM) for 24 h, and then the cell morphology was determined using an optical microscope (200 ×). This experiment was repeated three times, and representative images were shown. IVM, ivermectin.

To study the impact of ivermectin on the cell morphology of colorectal cancer cells, we treated colorectal cancer cells SW480 and SW1116 with different concentrations of ivermectin and then observed the alteration of cell morphology under an optical microscope. After 24 h culture, the cell morphology changed significantly. For the SW480 cells, as the ivermectin concentration increased, the cells became more and more sparse. Especially at 20 μM, the cells lost their original shape, became rounded, and shrunk or floated in the medium (Figure 3). Consistent with IC_50_ of ivermectin, ivermectin had more sensitivity to SW1116 cells since ivermectin at 5 μM resulted in the cells shrunk; when the concentration of ivermectin was 10 μM, the cells became round, shrunk, and floated in the medium, and the concentration of ivermectin increased to 20 μM, most of the cells shed and floated in the culture medium (Figure 3). These results suggest that ivermectin could promote the death of colorectal cancer cells in a dose-dependent manner.

### Ivermectin Induces Apoptosis in Colorectal Cancer Cells

**FIGURE 4:**
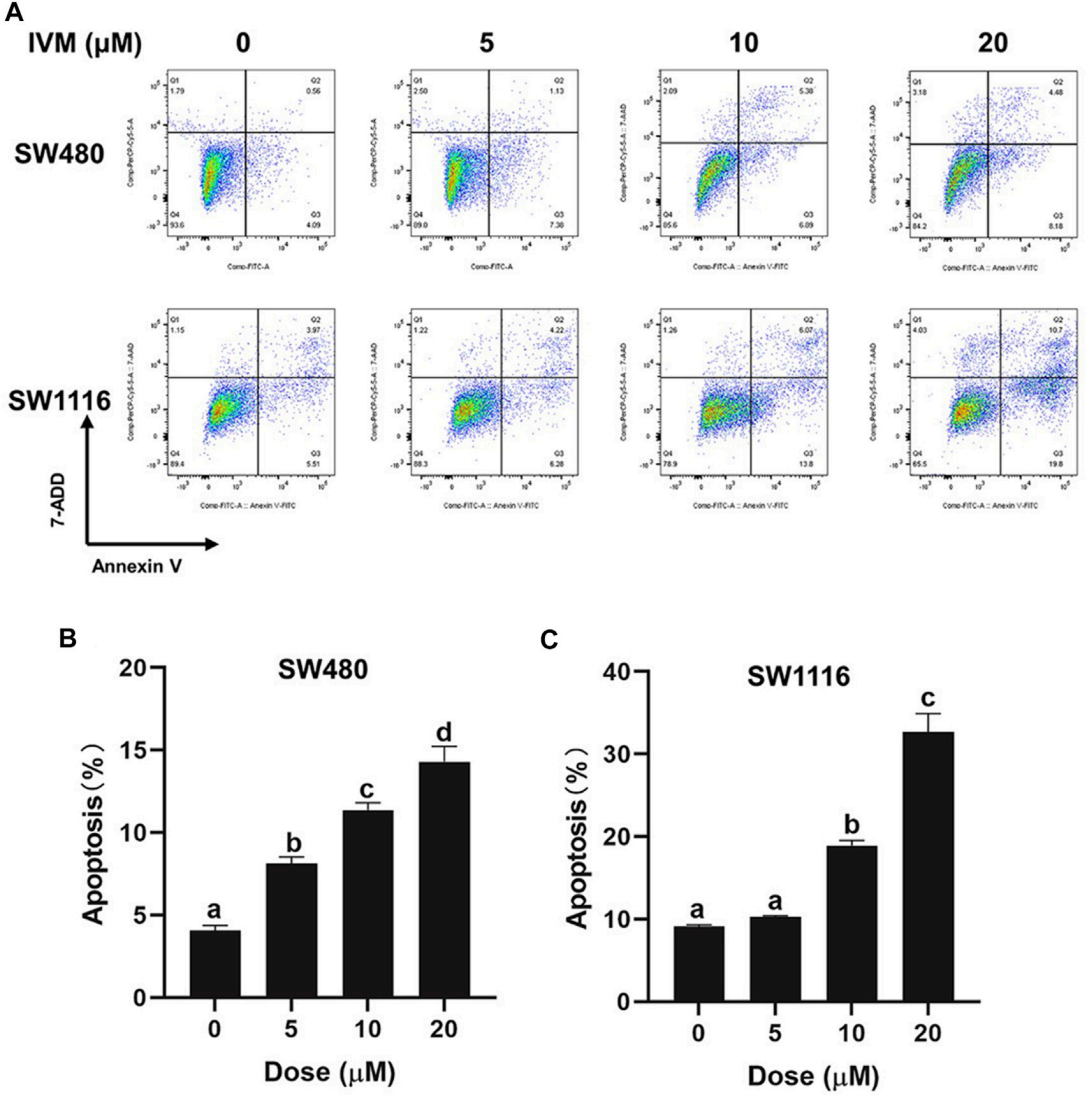
Ivermectin induced apoptosis in colorectal cancer cells. SW480 and SW1116 cells were treated with different concentrations of ivermectin (0, 5, 10, and 20 μM) for 6 h and then were stained, described in the “*Materials and Methods*” section. The total percentage of apoptosis is equal to the percentage of early apoptosis (Q2, Annexin V^+^7-AAD^-^) plus the percentage of late apoptosis (Q3, Annexin V^+^7-AAD^+^). Representative images from flow cytometry were shown in **(A)**. Data for SW480 **(B)** and SW480 **(C)** cells were summarized and analyzed using one-way ANOVA following Tukey’s *t*-test. The data are shown as the means ± S.E.M. of three independent experiments. (a–d): same letters, no statistical difference; different letters, the statistical difference (*p* < 0.05). IVM, ivermectin.

To determine whether ivermectin decreased the cell viability and the cell morphology of colorectal cancer cells via inducing cell apoptosis, we cultured colorectal cancer cells SW480 and SW1116 cells with indicated concentrations of ivermectin for 6 h, and apoptosis was evaluated by flow cytometry using Annexin V-FITC/7-AAD co-staining. As shown in Figure 4, ivermectin increased the proportion of apoptosis cells of SW1116 cells from 9.48% in the control group to 10.5%, 19.87%, and 30.5% in 5, 10, and 20 μM, respectively. Like this, ivermectin increased the proportion of apoptosis SW480 cells from 4.65% in the control cells to 8.51, 12.27, and 12.66% in 5, 10, and 20 μM, respectively. The results indicated that ivermectin had a dose-dependent effect on the induction of colorectal cancer cell apoptosis.

### Ivermectin Increases Caspase 3/7 Activity in SW480 and SW1116 Cells

**FIGURE 5:**
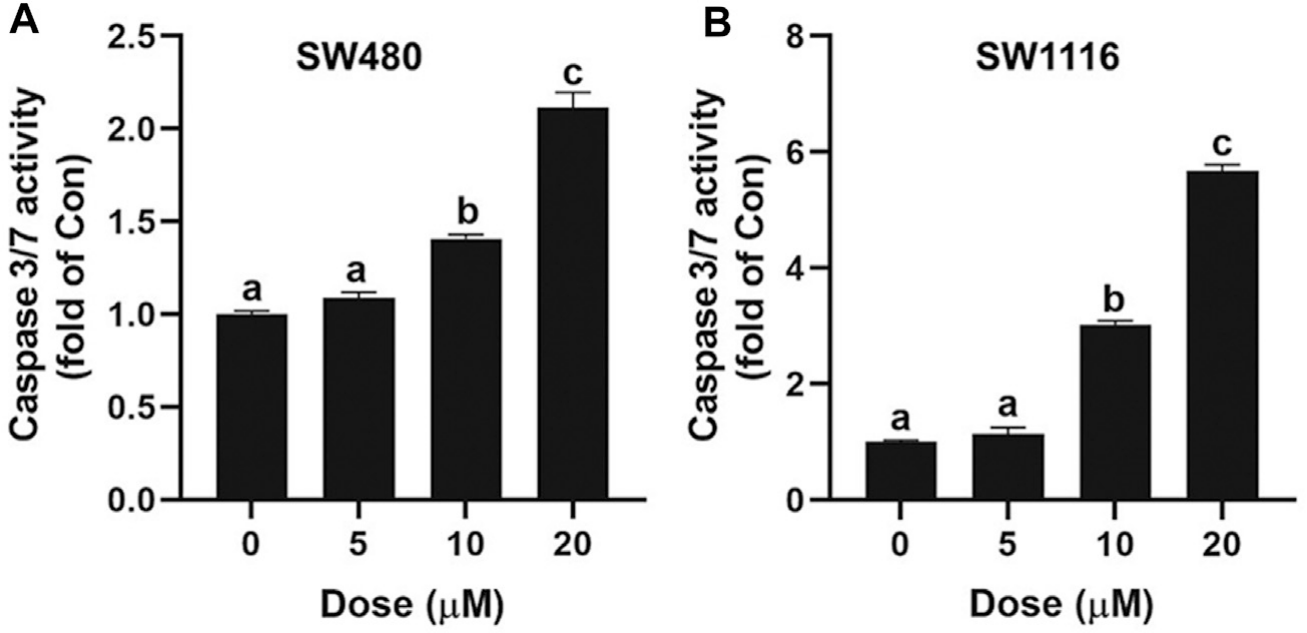
Effect of ivermectin on Caspase 3/7 in SW480 and SW1116 cells. SW480 **(A)** and SW1116 **(B)** cells were treated with different concentrations of ivermectin (0, 5, 10, and 20 μM) for 6 h, and then the Caspase 3/7 activity was measured described in the “*Materials and Methods*” section. Data were analyzed using one-way ANOVA following Tukey’s *t*-test. The data are shown as the means ± SD of three independent experiments. (a–c): same letters, no statistical difference; different letters, the statistical difference (*p* < 0.05). IVM, ivermectin.

Caspase-3 plays a vital role in the initiation of cell apoptosis. Caspase-3 typically exists in the cytoplasm in the form of zymogen (32KD). Caspase-3 activated by upstream signaling molecules can cleave the downstream key apoptosis proteins in the early stages of apoptosis and ultimately lead to apoptosis. In this study, the Caspase 3/7 Activity Apoptosis Assay kit was used to determine the effect of ivermectin on cell apoptosis. As shown in Figure 5, ivermectin increased Caspase 3/7 activity of SW480 and SW1116 cells in a dose-dependent manner.

### Ivermectin Affects the Expression of Apoptosis-Related Proteins in SW480 and SW1116 Cells

**FIGURE 6:**
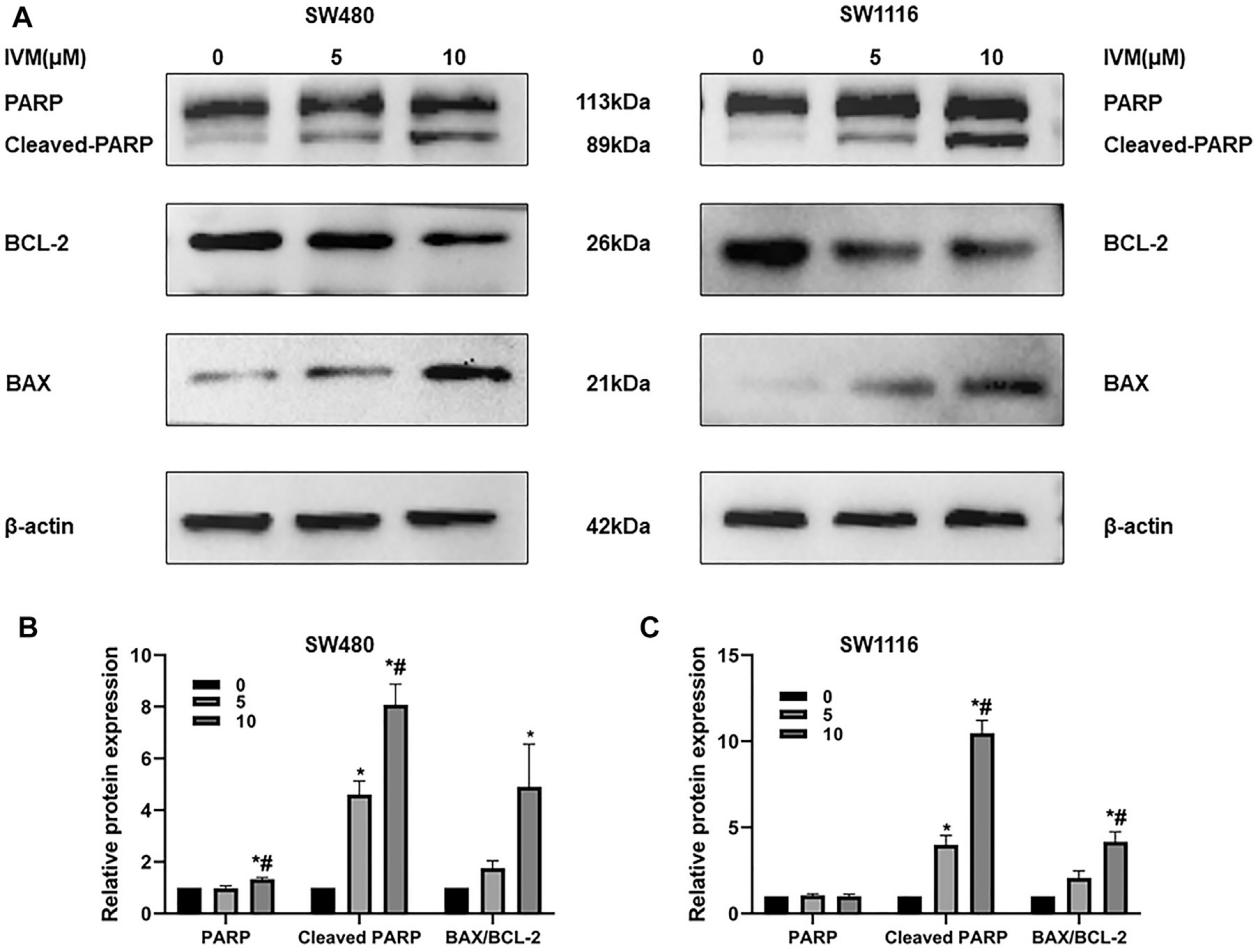
Influence of ivermectin on the Bax, Bcl-2, PARP, and Cleaved-PARP expression in SW480 and SW1116 cells. SW480 and SW1116 cells were treated with indicated concentrations of ivermectin (0, 5, and 10 μM) for 6 h and then were collected to determine the expression of apoptosis-related genes (Bax, Bcl-2, PARP, and Cleaved-PARP) by Western blot assay described as the “Materials and Methods” section. Representative gels for SW480 **(A, Left panel)** and SW1116 **(B, Right panel)** cells were shown, and data were summarized from three independent experiments **(B** and **C)**. The relative value was presented as fold induction to that of the control, which was normalized to β-actin. *p < 0.05, compared with the control group (0 µM); #*p* < 0.05, compared with the 5 µM group. IVM, ivermectin.

Bax and Bcl-2 are critical molecules in the endogenous apoptotic pathway. PARP (poly ADP-ribose polymerase) is a DNA repair enzyme, cleaved into Cleaved-PARP by the Caspase family protein, and cannot perform the repair function. The Western blot assay was used to determine the changes of apoptosis-related proteins Bax, Bcl-2, PARP, and Cleaved-PARP after treatment of colorectal cancer SW480 and SW1116 cells with ivermectin at the indicated doses. As the concentration of ivermectin increased, the expression of the proapoptotic protein Bax increased significantly, and the expression of the antiapoptotic protein Bcl-2 decreased; that is, the expression of the Bax/Bcl-2 ratio was gradually increasing (Figure 6). Also, the expression of Cleaved-PARP increased following the increase of ivermectin concentration (Figure 6).

### Ivermectin Increases Total and Mitochondrial ROS Generation in SW1116 Cells

**FIGURE 7:**
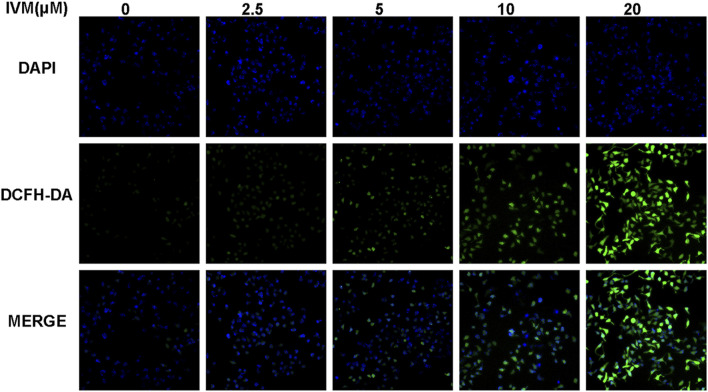
Ivermectin increased total intracellular ROS generation in SW1116 cells. SW1116 cells were treated with ivermectin at the indicated concentrations (0, 2.5, 5, 10, and 20 μM) for 6 h. Then the cells were co-stained by DCFH-DA and DAPI, described as the “*Materials and Methods*” section; cells were visualized using a fluorescence microscope. Representative images were shown, and the experiment was repeated three times. IVM, ivermectin.

**FIGURE 8:**
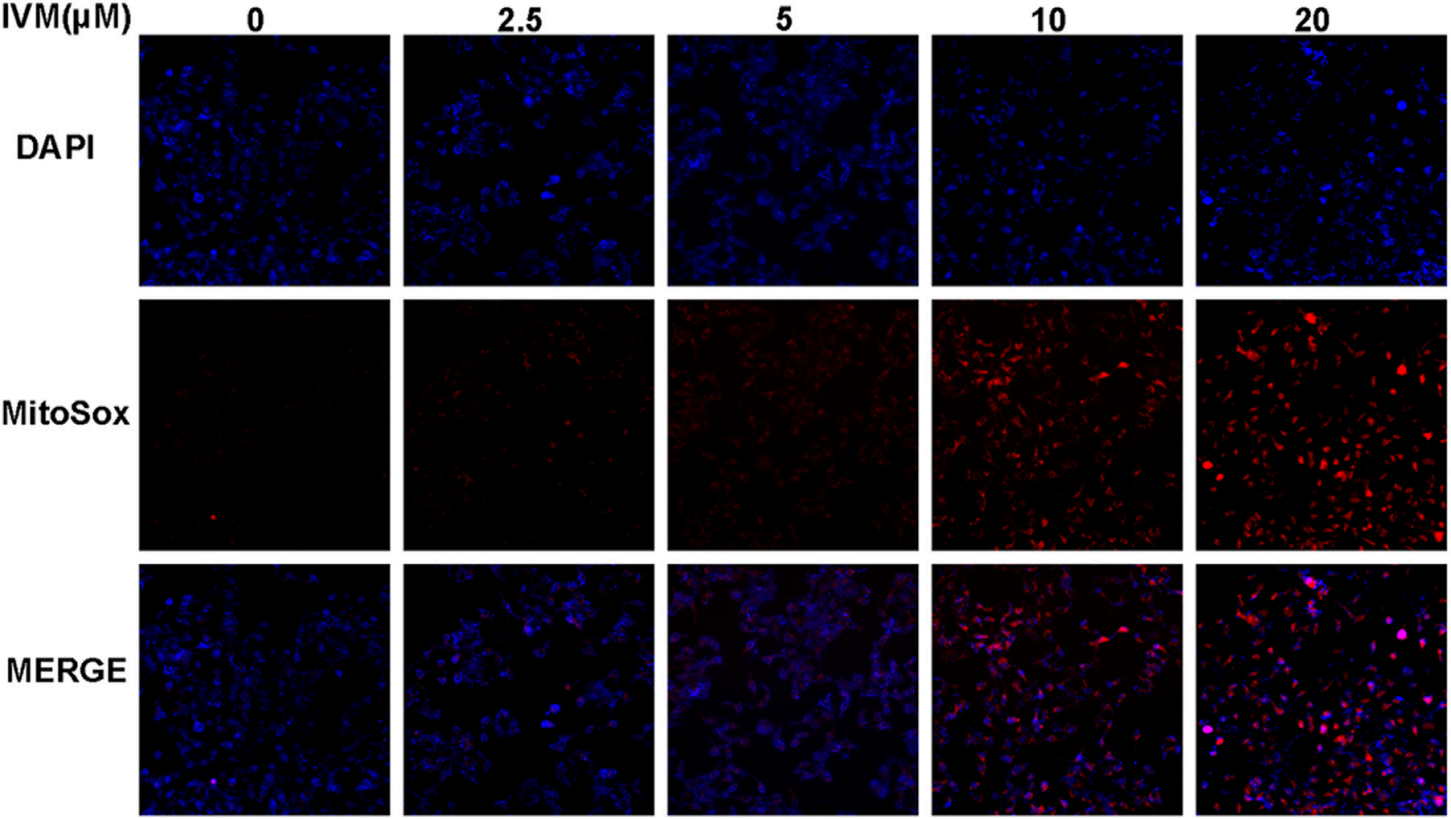
Ivermectin increased mitochondrial ROS generation in SW1116 cells. SW1116 cells were treated with ivermectin at the indicated concentrations (0, 2.5, 5, 10, and 20 μM) for 6 h. Then the cells were co-stained by MitoSOX and DAPI, described as the “*Materials and Methods*” section; cells were visualized using a fluorescence microscope. Representative images were shown, and the experiment was repeated three times. IVM, ivermectin.

To determine total intracellular total ROS and mitochondrial ROS levels, we treated SW1116 cells with ivermectin at the indicated concentrations for 6 h. After that, the cells were stained by DCFH-DA or MitoSOX with DAPI; cells were visualized using a fluorescence microscope. With the increase of the ivermectin concentration, the fluorescence of DCFA-DA (Figure 7) and MitoSOX (Figure 8) intensity gradually increased, indicating that ivermectin dose-dependently could increase the total and mitochondrial ROS content.

### NAC Reversed Ivermectin-Induced ROS and Cell Death in Colorectal Cancer Cells

**FIGURE 9:**
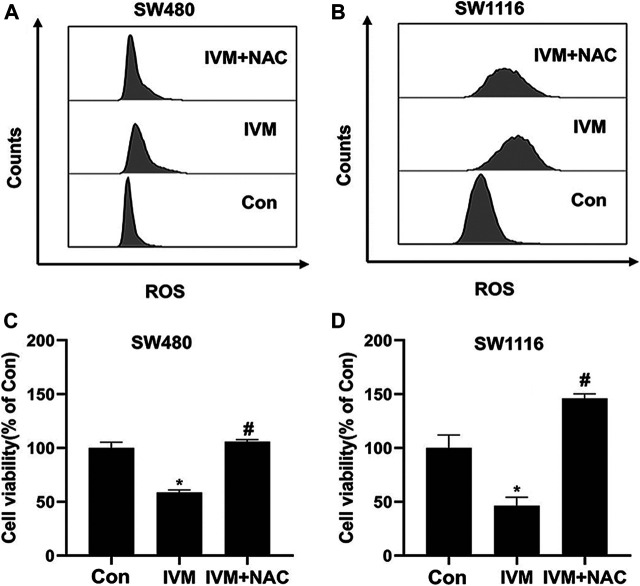
NAC reversed ivermectin-induced ROS and cell death in colorectal cancer cells. After SW480 **(A, C)** and SW1116 **(B, D)** cells were pretreated with ROS inhibitor NAC (5 mM) for 1 h and then were cultured in ivermectin (20 μM) for 6 h, they were stained by MitoSOX and detected by flow cytometry. The experiment was repeated three times, and representative histogram images were shown in **(A, B)**. **(C)** and **(D)**, after SW1116 and SW480 cells, were pretreated with NAC (5 mM) for 1 h and then were cultured in ivermectin (20 μM) for 6 h; the CCK-8 assay was performed to determine the cell viability described as the “*Material and Methods*” section. The experiment was performed three times with three biological replicates in each group. *p < 0.05, compared with the control group (0 µM).^#^ *p* < 0.05, compared with the IVM group. IVM, ivermectin; NAC, N-acetyl-l-cysteine.

To further illuminate the relationship between ROS and mitochondrial signal pathways in ivermectin-induced apoptosis, colorectal cancer cells were pretreated with ROS inhibitor NAC (5 mM) for 1 h and cultured in ivermectin (20 μM) for another 6 h. The cells were stained by MitoSOX and detected by flow cytometry. Compared with the control (Con, 0 µM) group, the peak shifted significantly to the right after treatment with ivermectin (20 µM); the peak shifted to the left after NAC administration (Figures 9A,B). This result illustrated that the NAC inhibited the ivermectin-induced ROS accumulation. Furthermore, this study used the CCK-8 kit to detected cell viability. For colorectal cancer cells SW480 (Figure 9C) and SW1116 (Figure 9D), compared with the control group without ivermectin, the cell viability decreased after ivermectin treatment could be reversed by administration of NAC.

### Ivermectin Induces Cell Cycle Arrest at S→G2/M in SW480 and SW1116 Cells

**FIGURE 10:**
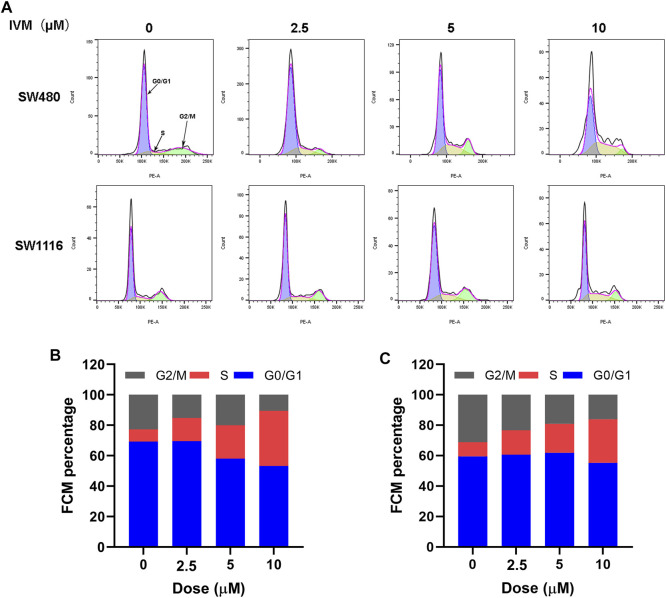
Ivermectin resulted in S-phase cell cycle arrest in a dose-dependent manner in colorectal cancer cells. **(A)** Different concentrations of ivermectin (0, 2.5, 5, 10, and 20 μM) were applied to SW480 and SW1116 cells for 12 h; flow cytometry was used to detect cell cycle changes. Representative cell cycle histograms were shown in **(A)**, and analysis of periodic distribution statistical results was shown in **(B)** and **(C)**. IVM, ivermectin.

The intercellular phase is divided into stationary phase (G0), early DNA synthesis phase (G1), DNA synthesis phase (S), and late DNA synthesis phase (G2/M). Propidium iodide (PI) single staining combined with flow cytometry could be used to detect the cell cycle changes after treating SW480 and SW1116 cells with different concentrations of ivermectin for 12 h. The fluorescent dye PI can bind to DNA in cells. The higher the DNA content, the more fluorescent dyes it binds, and the stronger the fluorescence intensity detected by flow cytometry. The cell cycle stages are divided, and the number of cells in different cycles is measured. Our results showed that the proportion of SW480 cells in the control group (0 µM) in the S phase is 5.31%. The proportion of S phase in each experimental group after 2.5, 5, and 10 μM ivermectin treatments were 25.2, 31.8, and 32.4%, showing an upward trend, indicating that ivermectin could block SW480 in the S phase, slow down the process of S phase entering G2/M phase, block cell growth, and division, and inhibit tumor cell proliferation (Figures 10A,B). It could also be seen from the same principle that the proportions of the S phase of SW1116 cells treated with 0, 2.5, 5, and 10 μM for 12 h were 15, 23, 29, and 37%, respectively (Figures 10A,C).

## Discussion

Even though the incidence of colorectal cancer has been declined for a long time, more than one-third of patients who underwent curative resection experience local and systemic recurrence (Wu et al., 2017). Therefore, colorectal cancer is still in an area of high unmet need for effective new treatments. Among different approaches, the new use of old drugs for colorectal cancer therapy is gaining importance. Ivermectin, a broadly-used old drug as an antiparasitic compound, is a drug candidate for repurposing as an anticancer drug (Alout and Foy, 2017). Accumulating evidence suggests that ivermectin has anticancer activities against breast cancer, digestive system cancer, urinary system cancer, hematological cancer, reproductive system cancer, brain glioma, respiratory system cancer, and Melanoma (Tang et al., 2021).

To evaluate the cytotoxicity of ivermectin on colorectal cancer cells, we used the CCK-8 assay to assess the effects of different concentrations of ivermectin on the viability of colorectal cancer cells. The results showed that ivermectin could effectively inhibit the proliferation of colorectal cancer cells in a concentration-dependence and time-dependence manner. There was a significant interaction between drug concentration and time of culture. After treatment with different concentrations, colorectal cancer cells showed inhibition of cell growth by optical microscopy.

Three pathways can lead to cell death, namely apoptosis, autophagy, and cell necrosis (D’Arcy, 2019). At present, targeting apoptosis is the most successful way to treat cancer in addition to surgery (Pfeffer and Singh, 2018). As an essential manner of cell death, apoptosis involves morphological changes with a series of stereotypy, DNA laddering fragments, phosphatidylserine (PS) eversion, mitochondrial transmembrane potential decreasing, and Caspase 3 activation (Kaczanowski, 2016; Majtnerová and Roušar, 2018). Therefore, we detected whether ivermectin is involved in the apoptosis of colorectal cancer cells by detecting these landmark events of apoptosis. Firstly, our study used Annexin V-FITC/7-AAD Apoptosis Kit to detect PS eversion. In normal cells, PS is mainly located on the cytoplasmic side of the plasma membrane. When apoptosis begins, PS transfers from the inside of the plasma membrane to the outside (Zhang et al., 2018b). Annexin V has a high affinity for PS and can bind to PS exposed to the external cellular environment. Our study used an Annexin V-FITC/7-AAD kit combined with flow cytometry to detect the effects of different concentrations of ivermectin on the apoptosis of colorectal cancer cells. As shown in the flow cytometry, ivermectin could induce the apoptosis rate of colorectal cancer cells in a dose-dependent manner which further confirmed the apoptosis effect of ivermectin. Secondly, our study used the Caspase 3/7 Apoptosis Kit to detect Caspase-3 activation. In the early stages of apoptosis, Caspase-3 is activated by upstream signal molecules. It can cleave the critical protein of apoptosis downstream and finally lead to cell apoptosis (Porter and Jänicke, 1999). This study showed that caspase3/7 activity increased with increasing ivermectin concentration.

As we all know, apoptosis pathways are mainly divided into extrinsic and intrinsic pathways (Carneiro and El-Deiry, 2020). Under the stimulation of drugs, the Bcl-2 family proteins regulate the permeability of the mitochondrial membrane change. Then cyt c is released into the cytoplasm, triggering the Caspase cascade reaction and finally leading to cell death (Elmore, 2007).

Considerable evidence indicates that both proapoptotic and antiapoptotic Bcl-2 family proteins were implicated in apoptosis induced by ivermectin-induced apoptosis. Song et al. (2019) revealed that ivermectin induced glioma cells apoptosis by upregulating the expressions of p53 and Bax, downregulating Bcl-2, activating cleaved caspase-3, and cleaved caspase-9. Zhang et al. (2019) observed that the ratio of Bax/Bcl-2 in the cytoplasm increased in ivermectin-induced apoptosis of Hela cells. In this study, as we expected, ivermectin inhibited apoptosis protein Bcl-2, increased the expression of apoptosis protein Bax and Cleaved PARP, suggesting that ivermectin may be involved in the intrinsic apoptotic pathway.

Intracellular ROS is involved in regulating apoptosis, cell cycle, and participating in various signal transduction pathways in cells (Yang et al., 2016). Deng et al. (2018) found that ivermectin can increase the content of intracellular ROS, and the increased ROS can activate the TFE3-dependent autophagy pathway. Similarly, Singh and Czaja (2007) found that ROS could ultimately induce hepatocyte death via caspase-dependent apoptotic pathways. This study used DCFH-DA and MitoSOX to detect the change of total ROS and mitochondrial ROS. As our results showed, ivermectin increased both total ROS and mitochondrial ROS in a dose-dependent manner. The flow cytometry results had shown that NAC could eliminate ROS induced by ivermectin to a certain extent. Next, the result of CCK-8 elucidated that NAC reversed ivermectin-induced cell death.

Cell cycle progression is one of the critical signaling mechanisms of homeostasis maintenance in healthy tissues and normal cells (Zhang et al., 2018a). Therefore, induction of cell arrest in cancer cells is a valuable strategy for anticancer drug development (Dominguez-Brauer et al., 2015). Several studies support that ivermectin leads to cell cycle arrest at G0/G1 phase in some cancer cells. For example, Song et al. (2019) found that ivermectin could block the cell cycle of C6 and U251 glioma cells at the G0/G1 phase. In addition, ivermectin could also cause cholangiocarcinoma cell arrest at the S phase (Intuyod et al., 2019). The present study demonstrated that ivermectin induced S-phase arrest in SW480 and SW1116 cells. Further characterization of ivermectin regarding CDK4 and cyclin A (S phase regulators) expression is needed to better characterize ivermectin’s effects upon the cell cycle.

**FIGURE 11:**
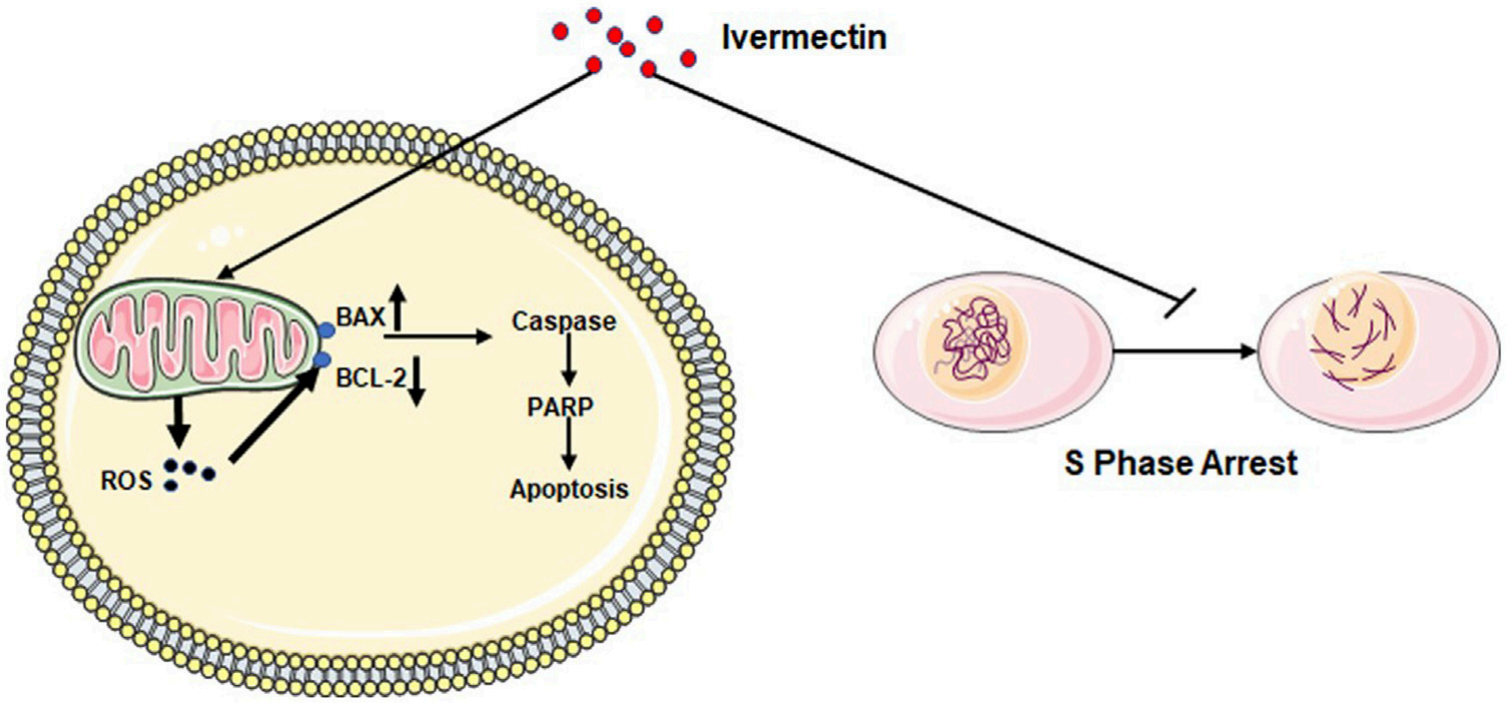
Diagram of the mechanism of ivermectin inhibiting the proliferation of colorectal cancer cells.

In conclusion, we have demonstrated that ivermectin may regulate the expression of crucial molecules Caspase-3, Bax, Bcl-2, PARP, and Cleaved-PARP in the apoptosis pathway by increasing ROS production and inhibiting the cell cycle in the S phase to inhibit colorectal cancer cells (Figure 11). Therefore, current results indicate that ivermectin might be a new potential anticancer drug for treating human colorectal cancer and other cancers.

## Data Availability

The original contributions presented in the study are included in the article/Supplementary Material, further inquiries can be directed to the corresponding authors.
