# Supplementary figures and images for: Ivermectin has New Application in Inhibiting Colorectal Cancer Cell Growth
Source: Front Pharmacol. 2021 Aug 13;12:717529. doi: 10.3389/fphar.2021.717529 (PMC8415024; doi:10.3389/fphar.2021.717529)

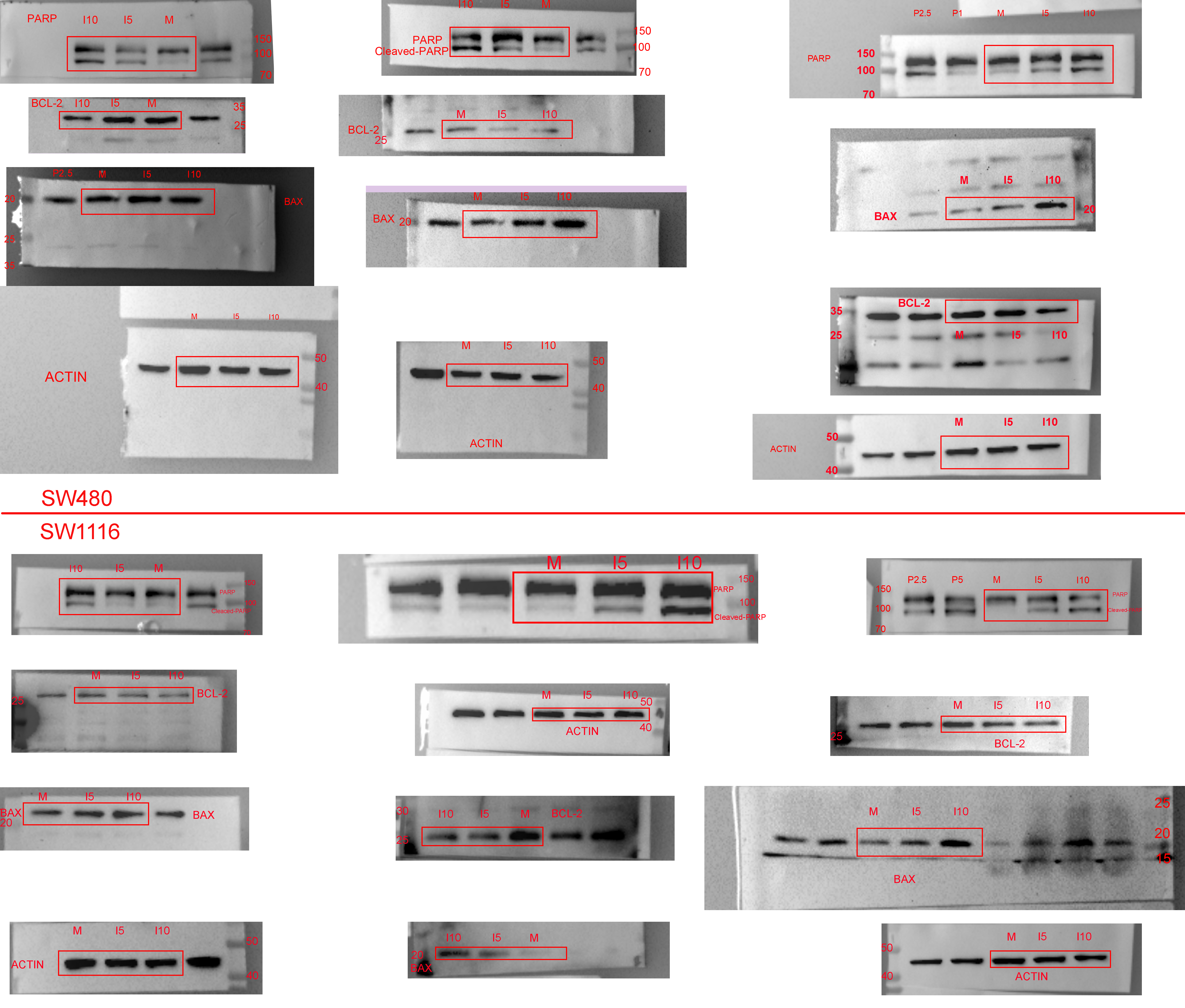

Supplement: Supplementary file 1 [file Image1.TIF]
